# *Bacillus subtilis* Improves Immunity and Disease Resistance in Rabbits

**DOI:** 10.3389/fimmu.2017.00354

**Published:** 2017-03-29

**Authors:** Mengjiao Guo, Fahao Wu, Guangen Hao, Qin Qi, Rong Li, Ning Li, Liangmeng Wei, Tongjie Chai

**Affiliations:** ^1^College of Veterinary Medicine, Shandong Agricultural University, Sino-German Cooperative Research Centre for Zoonosis of Animal Origin of Shandong Province, Tai’an City, China; ^2^Collaborative Innovation Center for the Origin and Control of Emerging Infectious Diseases, Taishan Medical University, Tai’an City, China; ^3^Tai’an City Central Hospital, Tai’an City, China

**Keywords:** probiotics, *Bacillus subtilis*, growth performance, innate immunity, disease resistance

## Abstract

Probiotics such as *Lactobacillus* and *Bifidobacterium* have been successfully used to promote growth and prevent diseases. Previous reports have demonstrated that *Bacillus subtilis* (*B. subtilis*) was a potential probiotic for animals. In this research, 180 *B. subtilis* were isolated from the soil, identified, and investigated *in vitro*. Furthermore, five *B. subtilis* were selected and mixed to investigate their effect on growth performance, immune response, intestine microbiota, and disease resistance in rabbits. Rabbits with a diet of 10^6^ CFU g^−1^ mixed *B. subtilis* exhibited the best growth performance and higher serum IgG and IgA than controls (*P* < 0.05). Moreover, dairy with *B. subtilis* can promote the balance of intestinal flora. The major proinflammatory factor and β-defensin were upregulated compared with the controls. After 7 weeks of feeding, the survival rate of the rabbits fed with *B. subtilis* was significantly higher than those in the control groups postinfected with *Escherichia coli*. At the same time, this study detected higher expression of β-defensin and reduced bacteria contents of the heart and cecal contents from the diet mixed with *B. subtilis* compared with the control groups. In conclusion, dietary supplementation with *B. subtilis* for rabbits could improve growth performance, intestinal homeostasis, and immune organ index and enhance innate immune response as well as disease resistance. These findings showed that the induction of β-defensin by *B. subtilis* might be an interesting new therapeutic strategy to strengthen innate defense mechanisms.

## Introduction

Antibiotics used in animal breeding introduce problems while improving growth performance (1). The administration of antibiotics, whether therapeutically or prophylactically, disturbs the normal microbiotic balance of the host (2). Consequently, an awareness of antimicrobial-resistant microorganisms caused by antibiotics encourages the development of probiotics (3). Probiotics are a safe alternative to antibiotics and are supplemented in the diet to prevent diseases and promote growth. Due to the spore’s resistance, survival in extreme environments, and long-term storage, spore-forming *Bacillus* spp. are considered to be suitable probiotics (4). They not only produce certain essential nutrients, such as amino acids, and vitamins K and B12 to promote growth performance (5) but they also promote the proliferation of beneficial anaerobic bacteria by consuming the free oxygen in the intestinal tract (6). In a previous study, *Bacillus subtilis* (*B. subtilis*) DJM-51 isolated from soil demonstrated strong antagonism toward the tomato pathogen *Clavibacter michiganense* subsp. *michiganense* ATCC 7429 (7).

According to guidelines for evaluating probiotics in food reported by a joint UN Food and Agricultural Organization World Health Organization working group, resistance to gastric acidity and bile salts are two of the most widely used *in vitro* tests based on both survival and growth studies. Furthermore, given serious concerns about increased resistance to antibiotics due to their regular use as additives, one aspect of probiotics that needs to be analyzed is antibiotic resistance. Probiotic strains, as well as bacteria used in food, can harbor resistant genes that can be transferred to pathogenic bacteria (8). As a result, probiotic strains intended for meat animals should be screened for antibiotic resistance (9).

The advantages of probiotics on hosts range from improved metabolism, immunostimulation, and anti-inflammatory status to the elimination of pathogens. Innate immunity is the first line of defense against the colonization of pathogens, and it plays a critical role in the pathogenesis and progression of intestinal disorders. The current findings confirmed that probiotics promote gut health through stimulation of the innate immune system in chronic Crohn’s disease-like ileitis mouse model (10). Recently, it was reported that dietary *B. subtilis* reduced clinical signs of enteric diseases, such as *Salmonella*, or clostridial diseases, such as necrotic enteritis, which correlated with increased innate immunity in broiler chickens (11, 12).

Several bacterial diseases afflict the rabbit breeding industry, including pathogenic *Escherichia coli* (*E. coli*) infection and rabbit *Clostridium perfringens* disease (13, 14). Treatment for the diseases cannot involve the use of long-term antibiotics due to their negative effects. For example, antibiotic-associated diarrhea is caused by *Clostridium* or *Staphylococcus aureus* (15). Probiotics, including potentially beneficial bacteria, enter the guts of humans and other animals, where their beneficial qualities improve intestinal balance, modulate and stimulate immune function, produce inhibitory compounds, and compete for chemicals and adhesion sites (16, 17). Many studies involving both pig and poultry diets with *B. subtilis* led to a reduction of *Clostridium* and *Coliforms* in host guts (18, 19). As an alternative, probiotics have shown considerable promise in promoting the health and performance of rabbits (20, 21).

In response, to identify potential candidate probiotics for rabbits, this study separated *B. subtilis* strains from the soil, screened for the strain with the best performance in an *in vitro* probiotic potential evaluation, and assessed that strain’s effects on weaning rabbits in a practical assessment.

## Materials and Methods

### *B. subtilis* and Pathogen

Soil samples were collected at a depth of 10 cm under the earth’s surface from Mount Tai, which is located in Tai’an city in the Shandong Province of China. First, 10 g of soil were suspended in 100 mL of sterile water in a conical flask. After incubation at 80°C for 1 h, the culture was diluted and spread on modified agar plates (glucose 5 g, yeast extract 5 g, peptone 5 g, beef paste 5 g, NaCl 5 g, and agar powder 20 g in 1 L of sterilized water with pH 7.0–7.2) and incubated at 37°C for 24–72 h. Suspected colonies were subjected to Gram stain and biochemical tests, including an arabinose biochemical tube test, glucose biochemical tube test, mannitol biochemical tube test, 3% NaCl gelatin biochemical tube test, and MR-VP biochemical tube test. Following those tests, 16S rRNA sequencing was performed with universal primers 27F (5′-AGAGTTTGATCCTGGCTCAG-3′) and 1492R (5′-GGTTACCTTGTTACGACTT-3′), and the sequences were blasted in NCBI.

The bacterial pathogen, enterohemorrhagic *E. coli*, was previously isolated from clinically infected rabbits developing acute diarrhea and stored by the Environmental Microbiology Laboratory at Shandong Agricultural University.

### Screening for Probiotic Properties *In Vitro*

#### Artificial Gastric Juice, Intestinal Juice, and Bile Salt Tolerance

This study was designed as in a model gastrointestinal tract experiment to investigate the survival of *B. subtilis*. Overnight cultures of *B. subtilis* were centrifuged at 3,000 × *g* for 10 min, washed twice with phosphate buffer saline, and resuspended in a new liquid medium (10^8^ CFU mL^−1^). The new media were adjusted to pH 2.5 by adding a hydrochloric acid solution with 1% pepsin or to pH 7.2 with 1% trypsin and incubated at 37°C. At 0, 1, and 2 h of incubation, 100 μL of the cultures were removed and spread on modified nutrition agar for cell number estimation. Respectively, the bacterial suspensions were inoculated into modified nutrition broth with 0.3% bile salt and cultured at 37°C for 24 h. Optical density at 600 nm (OD600) was measured and compared to a control culture without bile salts.

#### Protease Assay

These isolates were screened for neutral and alkaline protease production. Overnight cultures of *B. subtilis* strains were centrifuged at 6,000 × *g* for 15 min, and then the supernatant was assayed for protease activity according the SB/T 10317-1999 (22).

#### Antimicrobial Activities

The well diffusion method was performed on agar using cultured broth. Briefly, the target strain (10^6^ CFU mL^−1^) was incorporated into agar (1% w/v) plates, which were mixed for uniformity and poured onto plates to solidify. Overnight cultures of *B. subtilis* (10^8^ CFU mL^−1^) were transferred to holes (5-mm diameter) punched into the agar palates. The plates were then incubated as anaerobic or aerobic at 37°C for 24 h, depending on the target strain, and the antimicrobial ability was recorded as being in the inhibition zone (23).

#### Antibiotic Susceptibility

*Bacillus subtilis* suspensions were spread on Muller–Hinton agar plates, onto which antibiotic disks of penicillin, kanamycin, doxycycline, tetracycline, gentamicin, lincomycin, erythromycin, cefotaxime, and streptomycin were placed. After 24 h incubation at 37°C, the inhibition zone was measured (24).

### Animals and Cells

The healthy weaned New Zealand White rabbits (35 days) were kept in the same environment with sufficient room, feed, and ventilation. The rabbits were fed with a basal diet supplemented with 0, 10^5^, 10^6^, and 10^7^ CFU g^−1^ *B. subtilis* cells (mixture of BYS2, BQ3, BD17, BG5, and BGY12). The formula of the basic diet for each group is shown in Table 1. The rabbits were handled in accordance with the appropriate biosecurity guidelines, and all experimental protocols were approved by the Shandong Agricultural University Animal Care and Use Committee (no. SDAUA-2015-005).

**Table 1:** **Composition and nutrient levels of the experimental diet (air-dry basis)**.

Ingredients	Content (%)	Composition	Content
Soybean meal	5.00	CP (%)	16.04
Wheat bran	16.00	EE (%)	3.34
Alfalfa meal	6.00	CF (%)	16.73
Sunflower meal	9.00	Digestible energy (MJ/kg)^b^	10.32
Corn germ meal	24.00	Ca (%)	0.92
Corn	10.00	P (%)	0.42
Rice husk	6.00	Lys (%)	0.49
Soybean phospholipids	3.00	Met (%)	0.18
Barley husk	10.00		
Haulm powder	9.00		
Limestone	1.00		
Premix^a^	1.00		
Total	100		

*^a^Premix provided the following per kilogram of diets: VA, 8,000 IU; VD, 31,000 IU; VE, 50 mg; Lys, 1.5 g; Met, 1.5 g; Cu, 50 mg; Fe, 100 mg; Mn, 30 mg; Mg, 150 mg; I, 0.1 mg; Se, 0.1 mg*.

*^b^Calculated values*.

Rabbit kidney (RK-13) cells were grown in DMEM (GIBCO, Grand Island, USA) containing 10% fetal bovine serum (Transgen, Beijing, China) at 37°C in 5% CO_2_.

### 16S rRNA Gene Sequencing and Analysis

The cecal contents bacterial DNA of each sample was extracted using HiPure Stool DNA Kit B (Magen, Shanghai, China) following the manufacturer’s instructions. The DNA extractions were quantified by ultraviolet spectroscopy and amplified using universal primers of 341F (CCTACGGGNGGCWGCAG) and 806R (GGACTACHVGGGTATCTAAT) to target the V3–V4 domain of bacterial 16S rRNA. The amplicons were normalized, pooled, and sequenced on the Illumina GAIIx platform.

The raw Illumina fastq files were quality-filtered, de-multiplexed, and analyzed using quantitative insights into microbial ecology. Sequences with more than one ambiguous nucleotide or within correct barcodes or primers were removed. The Ribosomal Database Project classifier was used to classify tags into different taxonomies against Greengenes Database (version 20101006) with confidence threshold of 0.5. The software Mothur was used to cluster tags of more than 97% identity into operational taxonomic units (OTUs), and then the abundances of OTUs were calculated.

### Quantitative Real-time RT-PCR

Total RNA was extracted from the spleen and jejunum of rabbits using *Trans*Zol Up (Transgen, Beijing, China), and 1 µg total RNA was reverse-transcribed with HiScript^®^ II Q Select RT SuperMix for qPCR (+gDNA wiper) (Vazyme, Nanjing, China). Quantitative real-time PCR (qRT-PCR) oligonucleotide primers for immune-related genes and glyceraldehyde-3-phosphate dehydrogenase (GAPDH) were designed using Primer 3 software (http://bioinfo.ut.ee/primer3-0.4.0/), based on the published GenBank sequence (Table 2). qRT-PCR was performed using *TransStart* Tip Green qPCR SuperMix (Transgen Biotech Co., Ltd., Beijing, China) and the Applied Biosystems 7500 Fast Real-Time PCR System (Applied Biosystems, CA, USA). The qRT-PCR conducted in a total volume of 20 µL and the amplification steps consisted of 94°C for 30 s, 40 cycles of denaturation at 94°C for 5 s, and extension 60°C for 34 s, and a dissociation curve analysis. The purified PCR products were cloned into the pMD18-T and sequenced to verify correct amplification. Each sample was performed in triplicate. Data were calculated based on the 2^−ΔΔCt^ method. The relative expression mRNA was normalized to GAPDH.

**Table 2:** **Primers used in this study**.

Primer name	Sequence(5′-3′)	Product size (bp)	GenBank no.
NOD1 F	acaaggttcgcaaaatcctg	169	XM008261590.2
NOD1 R	ttgacaatggctttgctctg
NOD2 F	caacctcaagggcttctcag	241	XM008275042.2
NOD2 R	caggaaatgctgcaagatca
NLRC3 F	acatcatccgaggcaatctc	168	XM017338739.1
NLRC3 R	tctggtcctcagggaacatc
NLRX1 F	gtttcgggaggaggactacc	204	XM017338655.1
NLRX1 R	tccatgttcttgatgggaca		
IL-1β F	tggcacgtatgagctgaaag	115	NM001082201.1
IL-1β R	ggccacaggtatcttgtcgt		
IL-4 F	cactccggcagttctacctc	104	NM001163177.1
IL-4 R	gcagaggttcctgtcgagtc		
IL-8 F	ctctcttggcaaccttcctg	165	KT216053.1
IL-8 R	ttgcacagtgaggtccactc		
IL-10 F	aaaagctaaaagccccagga	143	NM001082045.1
IL-10 R	cgggagctgaggtatcagag		
IFN-γ F	ctcgaatttcggtggatgat	118	DQ680162.1
IFN-γ R	agcgtctgactcctttttcg		
PKR F	attggccattcatcatggtt	162	XM008258792.2
PKR R	ttctcggcagcatttctctt		
OAS F	gagctcctgaccatctacgc	182	XM002722126.3
OAS R	gccttgagctgtttcctgac		
DEFB114 F	taccagccacatgctctttg	134	XM017344866.1
DEFB114 R	cctgtcgacacagcaaatct		
DEFB124 F	gcaccaagcaagagtccttc	117	XM017342171.1
DEFB124 R	acgccagagccagctactta		
DEFB125 F	cgtgctgcatctccttaaca	114	XM008256168.2
DEFB125 R	gcgaagcagaaaattgatcc		
DEFB127 F	cccacagtaaccgagcaact	126	XM002710854.1
DEFB127 R	gctgaggcagcagtatctcc		
DEFB128 F	gggctcaaggctttctcttt	200	XM017342173.1
DEFB128 R	aaatctcgcctagcttgcac		
DEFB134 F	agcctgtctgcctggagtag	156	XM017337690.1
DEFB134 R	gatgaggagaggcttcatgg		
DEFB135 F	gctgcatctccaaatccaat	116	XM017337693.1
DEFB155 R	tagtgggatggtgcaactga		
NP 5 F	aggcaggcgtgttctgtact	154	M64602.1
NP 5 R	ggtctccacgcaaataagga		
Glyceraldehyde-3-phosphate dehydrogenase (GAPDH) F	aggtcatccacgaccacttc	202	NM001082253.1
GAPDH R	gtgagtttcccgttcagctc		

### Statistical Analysis

Statistical analyses were performed with SPSS 19.0, and one-way analysis of variance was used to identify differences among the groups. When variances were not homogeneous, the data were analyzed by the non-parametric Mann–Whitney *U* test or the Kruskal–Wallis test. The survival rate of rabbits was analyzed using the Kaplan–Meier method. Statistically significant differences required that *P* < 0.05.

## Results

### Probiotic Potential Evaluation of *B. subtilis In Vitro*

**Figure 1:**
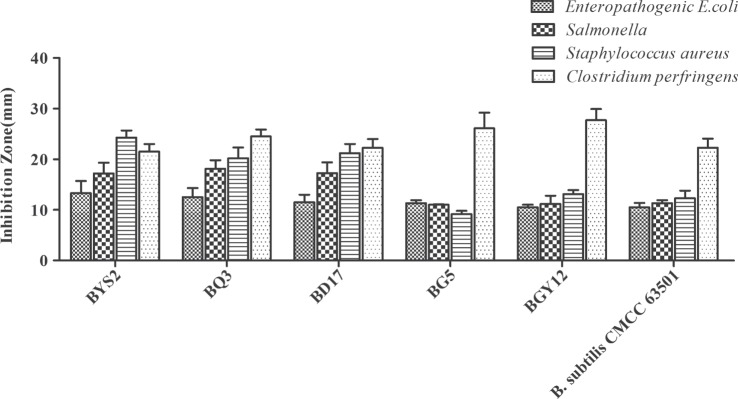
**Antibacterial activity of *Bacillus subtilis* against four pathogenic bacteria (*Enteropathogenic Escherichia coli* CVCC 1512, *Salmonella* CMCC 50094, *Staphylococcus aureus* ATCC 25923, and *Clostridium perfringens* NCTC 528)**. Bars represented the means ± SDs of three independent repetitions.

According to colony morphology and the results of microscopic examination, Gram-positive *Bacillus* strains with the typical volcano shape were selected as subjects for biochemical experiments. In total, 180 *B. subtilis* were isolated from the soil and confirmed by 16S rRNA sequencing. Five strains (BYS2, BQ3, BD17, BG5, and BGY12) exhibited excellent probiotic properties *in vitro*, including artificial gastric juice, intestinal juice, and bile salt tolerance (Table 3). BG5 and BGY12 had good performance in protease activities (Table 4). BYS2, BQ3, and BD17 showed good antimicrobial activities (Figure 1). All five *B. subtilis* were susceptible to the common antibiotics tested (data not shown). The 16S rRNA sequence of five *B. subtilis* was deposited in GenBank (Genbank no. KY750231-235).

**Table 3:** **Survival rate of *Bacillus subtilis* at artificial gastric juice, intestinal juice, and bile (%)**.

Strains	1 h		2 h		Bile

	Gastric juice	Intestinal juice	Gastric juice	Intestinal juice	
BYS2	100	100	100	100	47 ± 2.8
BQ3	100	100	100	100	49 ± 3.3
BGY12	100	100	100	100	100
BD17	81 ± 9.2	100	73 ± 4.3	100	48 ± 4.7
BG5	78 ± 4.3	100	82 ± 5.2	100	65 ± 4.0
*B. subtilis* CMCC63501	41 ± 3.1	100	17 ± 0.7	100	38 ± 1.8

*Values were expressed as means ± SDs of three independent repetitions*.

**Table 4:** **Neutral and alkaline protease production of *Bacillus subtilis***.

	BYS2	BQ3	BD17	BGY12	BG5	*B. subtilis* CMCC63501
Neutral protease (U mL^−1^)	1.3 ± 0.6	0	0	50.2 ± 3.8	60.7 ± 5.7	4.6 ± 1.3
Alkaline protease (U mL^−1^)	4.3 ± 1.6	2.3 ± 1.5	3.4 ± 0.9	48.6 ± 3.2	42.3 ± 5.1	2.5 ± 0.7

*Values were expressed as means ± SDs of three independent repetitions*.

### Growth Performance and Serum Immunoglobulin

**Figure 2:**
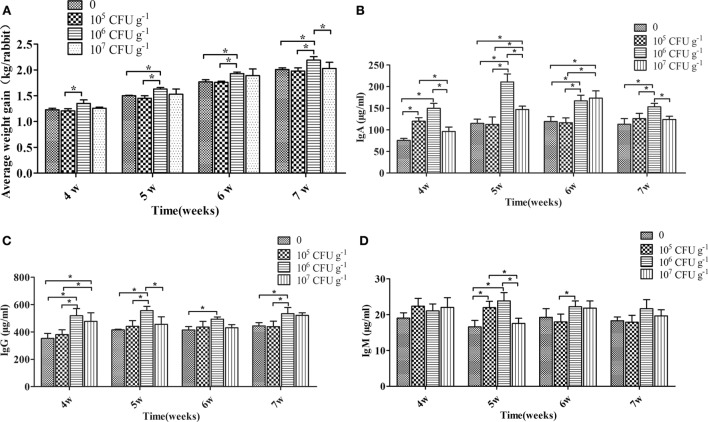
**Effects of dietary supplementation with *Bacillus subtilis* on growth performance and serum immunoglobulin in rabbits**. **(A)** The rabbits’ individual body weights were measured during feeding weeks 4–7. Bars were expressed as means ± SDs of three independent experiments (20 rabbits per experiment). One-way analysis of variance was conducted to examine differences. *P < 0.05. The blood samples were collected during feeding weeks 4–7. The concentrations of IgA **(B)**, IgG **(C)**, and IgM **(D)** are represented. Bars represented the means ± SDs of three independent experiments (five rabbits per experiment). Kruskal–Wallis test was conducted to examine differences. *P < 0.05.

After 4, 5, 6, and 7 weeks of feeding, rabbits fed with the diet of 10^6^ CFU g^−1^ *B. subtilis* showed the best growth performance and were heavier (*P* < 0.05) than the control diet rabbits. Rabbits fed a diet with 10^7^ CFU g^−1^ *B. subtilis* showed higher growth performance than the controls, but the difference was not significant (*P* > 0.05). However, no significant differences were observed between rabbits fed with 10^5^ CFU g^−1^ *B. subtilis* diets and the controls after 4, 5, 6, and 7 weeks of feeding (Figure 2A). After 4, 5, 6, and 7 weeks of feeding, rabbits fed with the diet of 10^6^ CFU g^−1^ *B. subtilis* had higher (*P* < 0.05) serum IgG and IgA than the controls. However, the serum IgA of rabbits fed with 10^7^ CFU g^−1^ *B. subtilis* were significantly different (*P* < 0.05) than the controls after only 5 and 6 weeks of feeding (Figures 2B,C). Consistent with the growth performance results, the serum immunoglobulin of rabbits fed 10^5^ CFU g^−1^ *B. subtilis* were virtually unchanged, especially IgM (Figure 2D). Based on these results research above, rabbits fed with the diet of 10^6^ CFU g^−1^ *B. subtilis* were chosen for the following assays.

### Immune Organ Index

**Figure 3:**
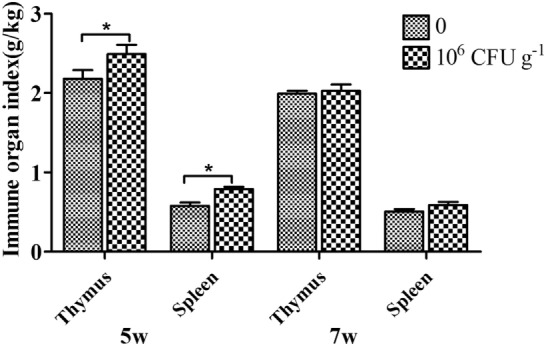
**Effects of dietary supplementation with *Bacillus. subtilis* on immune organ index in rabbits**. The thymus and spleen were weighed to calculate the immune organ index. Bars represented the means ± SDs of three independent experiments (five rabbits per experiment). Mann–Whitney *U* test was conducted to examine differences. *P < 0.05.

As shown in Figure 3, the relative weights of the thymus and spleen were significantly increased by diet with *B. subtilis* after 5 weeks of feeding. However, there was no difference between the group supplemented with *B. subtilis* and the controls after 7 weeks of feeding.

### Taxonomic Composition of Intestine Microbiota

**Figure 4:**
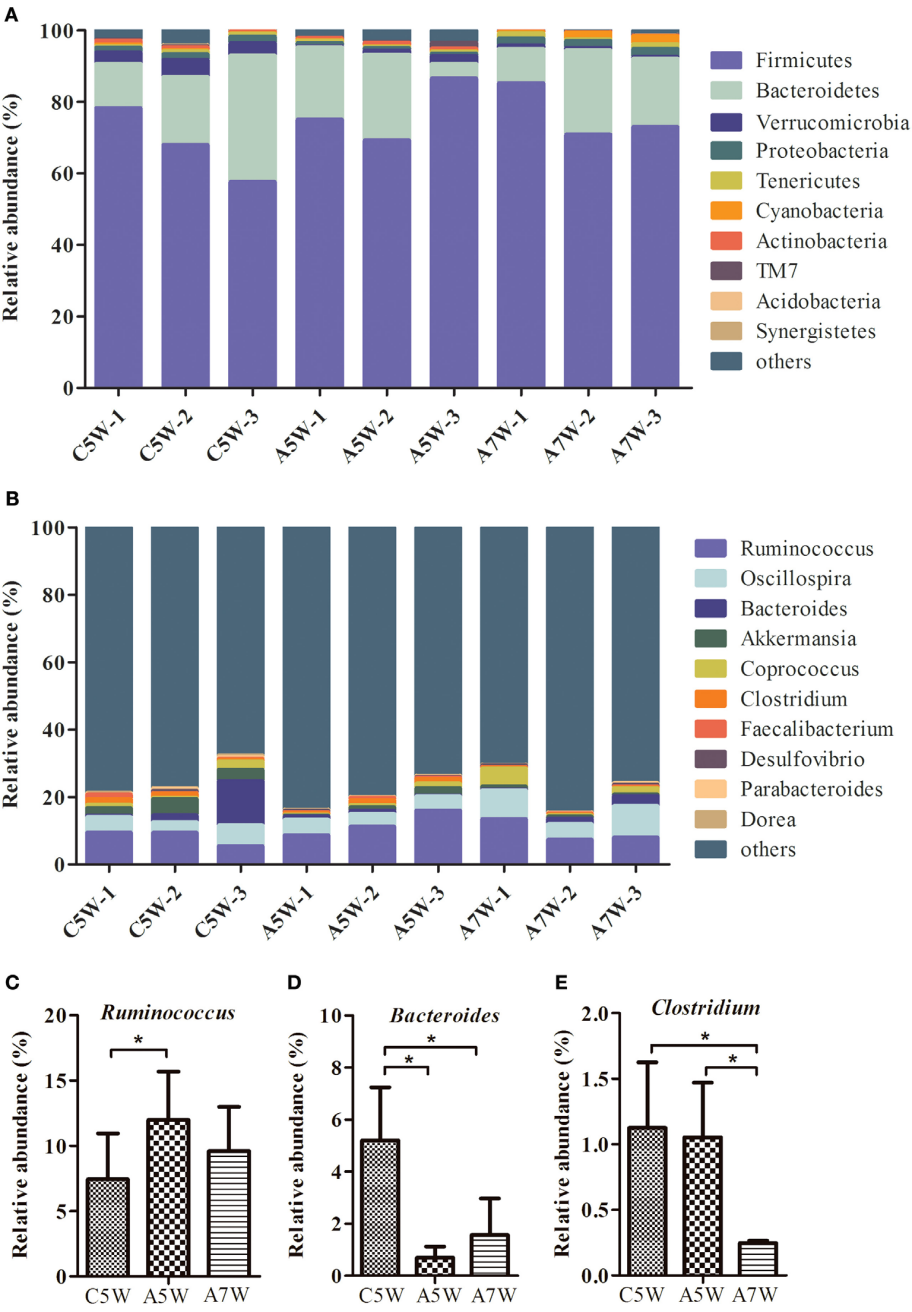
**Comparison of identified bacteria relative abundance in cecal contents**. The cecal contents of rabbits fed with 10^6^ CFU g^−1^ *Bacillus subtilis* diets and the controls were collected for high-throughput sequence analysis after 5 and 7 weeks of feeding. **(A)** Relative abundance at the phylum level. **(B)** Relative abundance at the genus level. **(C)** Relative abundance of three genera *Ruminococcus*, **(D)** *Bacteroides*, and **(E)** *Clostridium*. Bars were expressed as means ± SDs (*n* = 3). C5W, A5W, and A7W represented sample of control, active at 5 weeks, and active at 7 weeks of feeding, respectively. Kruskal–Wallis test was conducted to examine differences. *P < 0.05.

High-throughput sequence analysis of bacterial 16S rRNA V3–V4 region was conducted on cecal contents of rabbits after 5 and 7 weeks of feeding. As shown in Figure 4A, the 95.0–96.5% reads belong to Firmicutes, Bacteroidetes, Verrucomicrobia, and Proteobacteria at the phylum level. The relative abundance of the phyla *Firmicutes* was increased after feeding *B. subtilis*. The shifts in bacterial composition at the phylum level were caused by the changes at the genus level (Figure 4B). In particular, the relative abundance of *Ruminococcus* (Figure 4C; *P* < 0.05) was increased after 5 weeks of feeding. The percentage of *Bacteroides* (Figure 4D; *P* < 0.05) and *Clostridium* (Figure 4E; *P* < 0.05) were significantly decreased after 5 and 7 weeks of feeding.

### Expression of Innate Immune-Related Genes *In Vivo* and *In Vitro*

**Figure 5:**
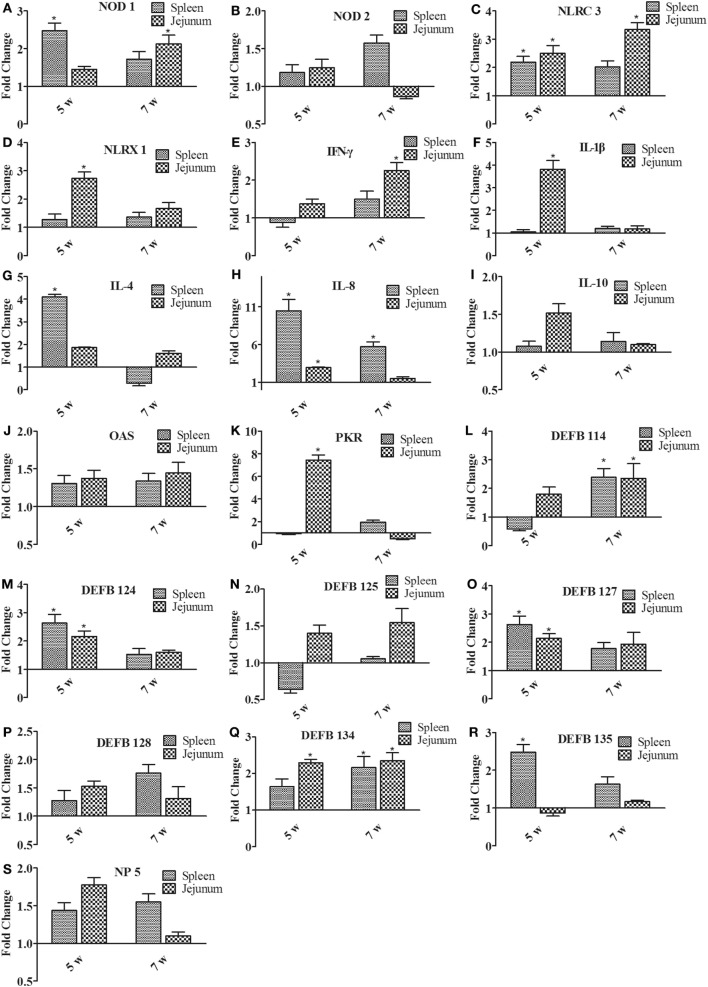
**The expression of immune-related genes in the spleen and jejunum of rabbits after 5 and 7 weeks of feeding**. **(A)** NOD1, **(B)** NOD2, **(C)** NLRC3, **(D)** NLRX1, **(E)** IFN-γ, **(F)** IL-1β, **(G)** IL-4, **(H)** IL-8, **(I)** IL-10, **(J)** OAS, **(K)** PKR, **(L)** DEFB114, **(M)** DEFB124, **(N)** DEFB125, **(O)** DEFB127, **(P)** DEFB128, **(Q)** DEFB134, **(R)** DEFB135, and **(S)** NP5. The fold change represents gene expression in diet with *Bacillus subtilis* compared to that of the controls. Bars represented the means ± SDs of three independent experiments (five rabbits per experiment). Mann–Whitney *U* test was conducted to examine differences. *P < 0.05.

To determine the induction of innate immunity in rabbits fed with a diet of *B. subtilis*, the expressions of innate immune-related genes were examined in the spleen and jejunum of rabbits fed with the probiotic compared to the controls after 5 and 7 weeks of feeding. Figure 5A shows that expressions of nucleotide oligomerization domain (NOD) 1 were upregulated in the spleen and jejunum. After 5 weeks of feeding, it was upregulated by 2.48-fold in the spleen (*P* < 0.05). The expression of NOD-like receptor (NLR) C3 and NLRX1 was generally higher in the jejunum than in the spleen (Figures 5C,D). For example, the expression of NLRC3 was elevated by 3.74-fold in the jejunum after 7 weeks of feeding (*P* < 0.05; Figure 5C), which is 2 times higher than that in the spleen. However, the NOD 2 expression was not obviously changed (Figure 5B). The expression of proinflammatory cytokines (IL-1β and IL-8) was upregulated in the spleen and jejunum (Figures 5F,H), while anti-inflammatory cytokines (IL-4 and IL-10) showed slight variation (Figures 5G,I). In particular, the expression of IL-8 was upregulated by 10.46- and 5.73-fold in the spleen after 5 and 7 weeks of feeding (*P* < 0.05; Figure 5H). Figure 5E shows that the expression of IFN-γ was upregulated by 2.46-fold (*P* < 0.05) in the jejunum after 7 weeks of feeding. The antiviral proteins OAS showed no significant difference (*P* > 0.05; Figure 5J), whereas the expression of PKR in jejunum was upregulated by 7.45-fold after 5 weeks of feeding (*P* < 0.05; Figure 5K). When the expressions of β-defensin (DEFB 114, DEFB 124, DEFB 125, DEFB 127, DEFB 128, DEFB 134, and DEFB 135) and α-defensin (NP 5) were investigated, all of these genes showed elevated expression in the spleen and jejunum after 7 weeks of feeding. The expressions of DEFB 114 and DEFB 134 were upregulated by two fold to three fold in the spleen and jejunum (*P* < 0.05; Figures 5L,Q). Moreover, the expressions of DEFB 124, DEFB 127, and DEFB 134 were upregulated in the spleen and jejunum after 5 weeks of feeding (*P* < 0.05; Figures 5M,O,Q). However, the expression of DEFB 125, DEFB 128, DEFB 135, and NP 5 showed no significant difference (*P* > 0.05; Figures 5N,P,R,S).

**Figure 6:**
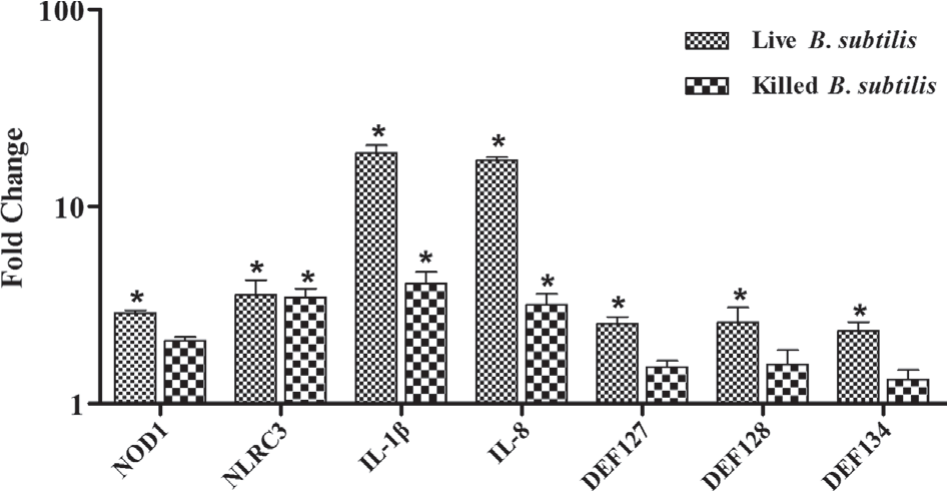
**Rabbit kidney (RK-13) cells were cocultured with live *Bacillus subtilis* or killed cells in DMEM without antibiotic for 2 h at 37°C**. The medium was removed, and cells were washed three times with PBS containing 1% penicillin–streptomycin solution to kill *B. subtilis*. RK-13 cells were cultured in DMEM containing 1% penicillin–streptomycin solution for 24 h. Also, 100 μl of the DMEM were plated onto nutrient agar to ensure effective killing of *B. subtilis*. The fold change represents gene expression of RK-13 cells stimulated with live *B. subtilis* and killed cells compared to that of controls. Bars represented the means ± SDs of three independent experiments. Mann–Whitney *U* test was conducted to examine differences. *P < 0.05.

To further explore the induction of innate immunity by *B. subtilis*, the RK-13 cell were stimulated by live *B. subtilis* and killed cells. The expressions of IL-1β and IL-8 were upregulated by 18.6- and 17.2-fold, respectively, after stimulation with live *B. subtilis* (*P* < 0.05; Figure 6), which were significantly higher than stimulation with killed cells. Similarly, the expression of DEFB127, DEFB 128, and DEFB 134 upregulated by 2.5-, 2.6-, and 2.3-fold, respectively, after stimulation with live *B. subtilis* (*P* < 0.05), which were not obviously changed after stimulation with killed cells.

### Survival Rate

**Figure 7:**
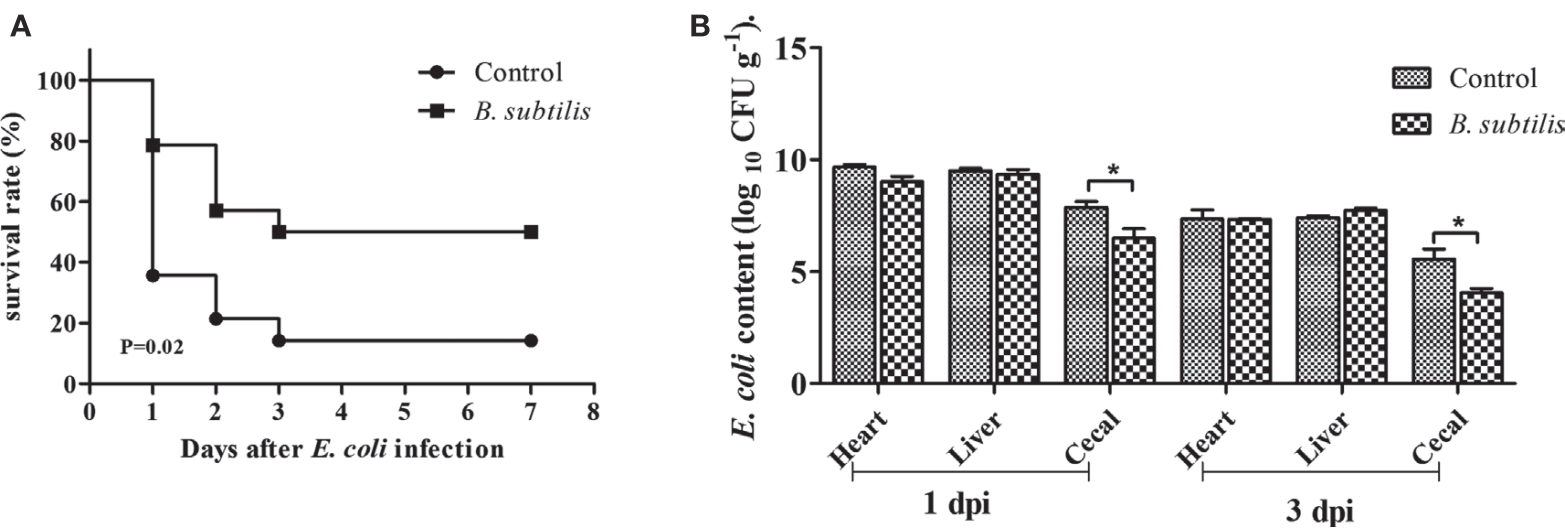
**Rabbits fed with the diet of 0 and 10^6^ CFU g−1 *Bacillus subtilis* were inoculated intraperitoneally with 1.5 mL of enterohemorrhagic *Escherichia coli* bacterial suspension (10^8^ CFU mL−1) after 7 weeks of feeding**. **(A)** The survival rate of rabbits after infection with *E. coli*: control and rabbits fed with the diet of 10^6^ CFU g^−1^ *B. subtilis* (*n* = 14). **(B)** *E. coli* content of infected rabbits at 1 and 3 dpi (log_10_ CFU g^−1^). Bars represented the means ± SDs of three independent experiments (five rabbits per experiment). Mann–Whitney *U* test was conducted to examine differences. *P < 0.05.

The survival rate of rabbits fed *B. subtilis* was significantly higher than the controls after the *E. coli* challenge. At 1, 2, and 3 days postinfection (dpi), the survival rate of rabbits fed *B. subtilis* was 79.2, 57.8, and 50.6%, respectively, which were higher than the control group (*P* < 0.05; Figure 7A).

### *E. coli* Content in *E. coli*-Infected Rabbits

As shown in Figure 7B, the number of *E. coli* in the heart and cecal contents in the *B. subtilis* group were declined compared to controls at 1 dpi. At 3 dpi, the number of *E. coli* in all tested tissues declined compared to those of 1 dpi. In particular, rabbits fed with the diet of *B. subtilis* had lower (*P* < 0.05) *E. coli* content in their cecal contents compared to the controls at 3 dpi.

### Expression of Innate Immune-Related Genes in the Spleen and Jejunum of the Infected Rabbits

**Figure 8:**
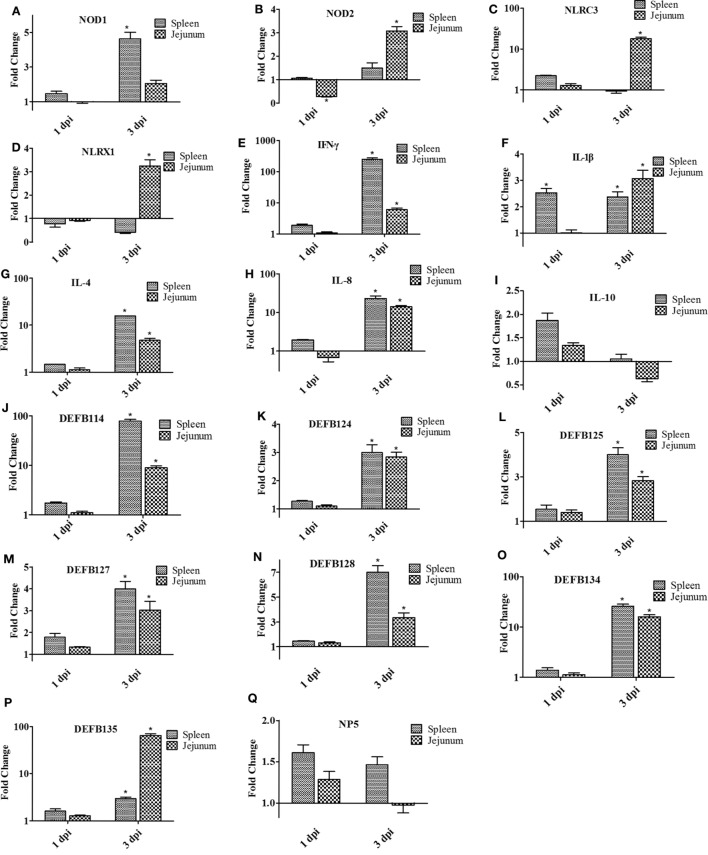
**The expression of immune-related genes in the spleen and jejunum of rabbits at 1 and 3 dpi**. **(A)** NOD1, **(B)** NOD2, **(C)** NLRC3, **(D)** NLRX1, **(E)** IFN-γ, **(F)** IL-1β, **(G)** IL-4, **(H)** IL-8, **(I)** IL-10, **(J)** DEFB114, **(K)** DEFB124, **(L)** DEFB125, **(M)** DEFB127, **(N)** DEFB128, **(O)** DEFB134, **(P)** DEFB135, and **(Q)** NP5. The fold change represents gene expression in diet including *Bacillus subtilis* compared to that of controls. Bars represented the means ± SDs of three independent experiments (five rabbits per experiment). Mann–Whitney *U* test was conducted to examine differences. *P < 0.05.

As shown in Figure 8, the expression of all innate immune-related genes was not as obvious as in the spleen and jejunum at 1 dpi. However, the expression of NOD2, NLRC3, and NLRX1 in the jejunum was significantly upregulated at 3 dpi. The expression of NLRC3, in particular, was upregulated by 18.18-fold (*P* < 0.05; Figure 8C), whereas the expression of NOD1 increased by 4.63-fold in the spleen (*P* < 0.05; Figure 8A). Similarly, the expression of IL-1β, IL-4, IL-8, and IFN-γ in the spleen and jejunum showed upregulating tendencies, but to a greater extent. At 3 dpi, the expression of IFN-γ increased 251.41-fold (*P* < 0.05; Figure 8E). Furthermore, the expression level of β-defensin was generally higher in the spleen than in the jejunum at 3 dpi. DEFB 124,DEFB 125,DEFB 127, and DEFB 128 showed a relatively low expression compared with DEF114 and DEFB134, with a fold change between twofold and eightfold in the spleen or the jejunum. At 3 dpi, the expressions of DEFB 114 and DEFB 134 were upregulated and reached 78.93- and 25.89-fold (*P* < 0.05; Figures 8J,O). However, in the jejunum, the expression of DEFB 135 was upregulated by 65.69-fold at 3 dpi (*P* < 0.05; Figure 8P).

## Discussion

To influence the gastrointestinal tract, probiotics should also be excellent in adapting to acidic conditions in the stomach and in bile salt in the duodenum. Hydrochloric acid in gastric juice can kill bacteria in food to ensure the safety of the stomach and intestines and activate pepsin for digestion. It has also been reported that good bile tolerance benefits colonization in the host’s gastrointestinal tract (25). Maintaining a high survival rate in acid and bile salt tolerance tests means that an effective number of live bacteria will enter the intestinal tracts and successfully play a role. After overnight incubation, the *Bacillus* cultures were examined microscopically, and no more than 5% of free spores were observed. Therefore, the remarkable resistance of five *B. subtilis* strains to gastric intestinal juice and bile salt conditions can not be due to the presence of spores. *Bacillus* sp. can produce certain essential nutrients and extracellular enzymes, as well as provide necessary growth factors to promote host growth (5). BG5 and BGY12 had good performance in the protease activities. BYS2, BQ3, and BD17 appeared to inhibit pathogenic bacteria, and, of course, all five strains showed no antibiotic resistance. On the other hand, strain that does not have any probiotic activity or killed cells can not produce digestive enzymes, vitamins, and antibacterial substances, which were produced by probiotic. Based on these reasons, we speculated that strains without probiotic activity and killed cells have no significant effects on growth performance, intestinal flora and disease resistance.

In poultry and pigs, *B. subtilis* (1.8 × 10^6^ CFU g^−1^ diet) promoted animal growth and immune regulation (18, 26) and can also help animals resist intestinal pathogenic bacteria infection (27). In a similar study, *Bacillus circulans* (2 × 10^6^ CFU g^−1^ diet) significantly enhanced the growth performance and non-specific immunity levels and disease resistance of fish (28). In the present study, rabbits fed with a diet containing *B. subtilis* at 10^6^ CFU g^−1^ had significantly improved average weight gain, which might result from the production of nutrients and digestive enzymes. Rabbits fed a diet of 10^7^ CFU g^−1^ *B. subtilis* did not grow as well as those fed with 10^6^ CFU g^−1^, perhaps because nutrients in the gut of rabbits was absorbed by the addition of *B. subtilis*. In previous research, there was no difference between the group supplemented with *B. subtilis* alone and the controls (29). Such discrepancies could stem from differences among experimental conditions or among species of probiotics.

In this study, dairy with *B. subtilis* significantly improved concentrations of the serum immunoglobulins IgG and IgA of rabbits. Moreover, the relative weights of the spleen were significantly increased by dietary supplementation with *B. subtilis* after 5 weeks of feeding. It can thus be deduced that *B. subtilis* might improve immune function, which has been reported to reduce weaning stress and improve the growth performance of rabbits at weaning (30). It has been reported that *Bacillus cereus* increased the concentrations of beneficial bacteria and decreased the concentration of harmful bacteria (31). In the current study, the relative abundance of *Bacteroides* and *Clostridium* were significantly decreased, and *Ruminococcus* increased after feeding *B. subtilis*. The *Ruminococcus* contributed to the digestion and absorption of fiber in herbivores (32). The increase of *Ruminococcus* may promote the growth performance of rabbits. In addition, the increase of *Bacteroides* and *Clostridium* in the intestine leads to colitis and carcinogenesis (33). These findings indicate that dairy with *B. subtilis* can regulate intestinal flora and maintain intestinal health.

The innate immune system is used as the first line of defense to various pathogens. NLRs are a kind of pattern recognition receptor (PRR). PRRs have been implicated in pathogen control and elimination and have been recently identified as key mediators of inflammatory and immune responses. There are few reports about NLRs in rabbits, and only the predicted sequences are available in Genebank. At present, the full lengths of some NLRs in rabbits have been obtained, and their biological functions (no published data) have been verified. In this study, the dietary administration of *B. subtilis* increased the NOD1, NLRC3, and NLRX1. NLRs detect and sense microbial components and then lead to the activation of an inflammatory response and promote the secretion of cytokines (34, 35). A previous study has shown that NLRCs induce IFN-γ and IL-1β gene expression in Japanese flounder (36). Olive flounder NOD1 not only plays a major role in inhibition of *Edwardsiella tarda* growth but also enhances the activation of IL-1β, which is in turn implicated in the initiation of inflammation and host defense (37). IL-1β is a major proinflammatory cytokine that induces its own expression as well as the expression of other proinflammatory cytokines, adhesion molecules, and chemokines such as IL-6, IL-8, and TNF-α, in turn initiating an inflammatory response and recruitment of antimicrobial cells such as neutrophils and macrophages (38). The main biological activity of IFN-γ is immune regulation, which includes innate and adaptive immunity. In previous studies, pretreatment with *B. subtilis*, the IFN-γ transcript levels remained high after infection with *Citrobacter rodentium* (39, 40). It has been shown to be a proinflammatory factor and has an antiviral effect (41). In this study, IFN-γ, IL-1β, and IL-8 were upregulated by dietary *B. subtilis*. It may be due to the activation of NLRs.

Previous studies have suggested that several probiotics induce innate immunity through β-defensin 2 induction in Caco-2 cells (42). The antimicrobial peptide (AMP) is a class of molecules that occurs in all forms of life, from multicellular organisms to bacterial cells. In higher organisms, AMPs contribute to innate immunity and are part of the first line of defense against various microorganisms, including Gram-positive and Gram-negative bacteria, fungi, and viruses (43). The defensins are small, cationic, cystine-rich AMPs with broad antimicrobial activities. They can recruit immature dendritic cells and macrophages to reach the skin and mucosal tissues of microbial invasion through chemokine receptors, thus inducing specific immunity of pathogenic bacteria. In mammals, defensins are divided into α-, β-, and θ-defensins (44). The results of the present study suggest that the α-defensin and β-defensin were induced by a diet with *B. subtilis*. On the one hand, defensins can directly act on pathogenic microorganisms and more importantly participate in the innate immunity as well as the initiation and regulation of adaptive immune response. Previous study has demonstrated that lactobacilli and the VSL#3 bacterial mixture restored intestinal barrier function through the upregulation of human-beta defensin 2 *via* induction of proinflammatory pathways of the nuclear factor-κB (45). However, the genomic structure and PGN of bacteria also exist in the killed cells. They can be identified by the host PRRs, which trigger the innate immune response. So killed cells are able to induce a certain degree of protective immune responses. Our results indicated that live *Bacillus* showed stronger ability in induce innate immunity than the killed cells when used the same dose in the RK-13 cells. To the best of our knowledge, this research is the first report on the innate immune response to *B. subtilis* in rabbits.

In return, the survival rate of rabbits fed *B. subtilis* was higher than that of the controls after they were challenged with *E. coli*. This conclusion was supported by increased body weights, serum immunoglobulin, and immune organ index and enhanced expression of major innate immunity genes involved in initiating and regulating immune response against *E. coli*. It is worth noting that β-defensin was significantly upregulated in the *B. subtilis* feeding group postinfection with *E. coli*, especially DEFB 114, DEFB 134, and DEFB 135. Given that β-defensin has a strong antibacterial ability, the higher expression of β-defensin may be the most important reason for disease resistance. Meanwhile, the present results showed that the number of *E. coli* in the cecal contents significantly declined with a diet of *B. subtilis* during the test days. As various reports have shown, several *bacilli* have established antimicrobial properties against various Gram-positive and Gram-negative pathogenic bacteria (46). In this study, BYS2, BQ3, and BD17 have also shown strong inhibition against *E. coli in vitro* and therefore possess potent immunostimulatory capacities. It is also important that the intestinal flora is mainly composed of anaerobic bacteria, and relatively few aerobic bacteria. *B. subtilis* can inhibit the growth of pathogenic *E. coli* by consuming oxygen in the intestine and regulating the balance of intestinal flora. Previous research showed that *B. subtilis* PY79^hr^ can inhibit colonization and persistence of *Salmonella enteritidis, C. perfringens*, and *E. coli* (47). In the intestine, *B. subtilis* may prevent the overgrowth of *E. coli*, enhance the resistance to *E. coli* invasion of the intestinal tract, and ameliorate disease processes. As a result, a diet including *B. subtilis* decreased the concentration of *E. coli in vivo*. *B. subtilis* may be competitive exclusion approaches in the control of infection with enteric pathogens.

In conclusion, the five *B. subtilis* that were selected for *in vitro* experiment showed good probiotic potential in rabbits. Enhancing the disease resistance of rabbits fed with *B. subtilis* may due to the probiotic’s influence on the improved growth performance, serum immunoglobulin, immune organ index, intestinal homeostasis, and immune response of rabbits, as well as its antibacterial benefits. The data from this study enhance the understanding of the mechanism of action between probiotics and the host, allowing for the development of probiotic-based strategies to prevent pathogenic infection.

## Author Contributions

MG and FW designed and conducted the study, performed most of the experiments, and wrote the manuscript. GH and RL performed the calculation with support from QQ. FW and NL collected samples. TC and LW discussed the results and revised the manuscript.

## Conflict of Interest Statement

The authors declare that the research was conducted in the absence of any commercial or financial relationships that could be construed as a potential conflict of interest.
